# Porcine alveolar macrophage CD163 abundance is a pivotal switch for porcine reproductive and respiratory syndrome virus infection

**DOI:** 10.18632/oncotarget.24040

**Published:** 2018-01-06

**Authors:** Tong-Yun Wang, Yong-Gang Liu, Liang Li, Gang Wang, Hai-Ming Wang, Hong-Liang Zhang, Shi-Fei Zhao, Jia-Cong Gao, Tong-Qing An, Zhi-Jun Tian, Yan-Dong Tang, Xue-Hui Cai

**Affiliations:** ^1^ State Key Laboratory of Veterinary Biotechnology, Harbin Veterinary Research Institute of Chinese Academy of Agricultural Sciences, Harbin 150001, China

**Keywords:** PRRSV, CD163, infection, switch, abundance

## Abstract

Porcine reproductive and respiratory syndrome virus (PRRSV) is a problematic virus that is difficult to control. The principal target cells for PRRSV infection are porcine alveolar macrophages (PAMs). Increasing evidence has demonstrated that CD163 is the determinant receptor for PRRSV infection. However, the relationship between CD163 abundance and PRRSV infection is unclear. In this study, we first generated primary immortalized PAMs (iPAMs) using SV40 large T antigen and demonstrated that CD163 expression is suppressed by the alternative splicing of mRNA in iPAMs. Two forms of CD163 transcripts were discovered, and most iPAMs expressed a short-form CD163 transcript that lacked from scavenger receptor cysteine-rich tandem repeat 1 (SRCR1) to SRCR5 of the functional domain. More importantly, using flow cytometric cell sorting technology, we isolated CD163-positive single-cell-derived clones with varying CD163 abundances to investigate the relationship between CD163 abundance and PRRSV infection. For the first time, we showed that cells with low CD163 abundance (approximately 20%) do not initiate PRRSV infection, while cells with moderate CD163 abundance display limited infection. PRRSV initiated efficient infection only in cells with high CD163 abundances. Our results demonstrate that CD163 abundance is a pivotal switch for PRRSV replication.

## INTRODUCTION

Porcine reproductive and respiratory syndrome virus (PRRSV) is a dangerous pathogen in the swine industry worldwide, especially with the emergence of highly pathogenic PRRSV [1, 2]. PRRSV is a positive-strand RNA virus with a length of approximately 15 kb that belongs to the Arteriviridae family [1]. According to recent taxonomic classifications, the Arteriviridae family includes three other viruses, namely, equine arteritis virus (EAV), lactate dehydrogenase-elevating virus (LDV) and simian hemorrhagic fever virus (SHFV). The cell tropism of arteriviruses is an interesting topic that was reviewed by Zhang et al.; except for EAV, which displays relatively broad cell tropism, the other member viruses in this family exhibit very limited cell tropism [3]. PRRSV infection is limited to porcine alveolar macrophages (PAMs), differentiated blood monocytes (BMos), dendritic cells (DCs) and a subset of bone marrow (BM) cells [4].

The cell tropism of PRRSV is largely dependent on host cell receptors. Currently, several putative receptors have been demonstrated to participate in PRRSV infection. These putative PRRSV receptors include CD163 [5], CD151 [6], CD169 [7], Heparin sulfate (HS) [3], vimentin [8], DC-SIGN (dendritic cell-specific intercellular adhesion molecule-3-grabbing non-integrin/CD209) [3] and MYH9 (non-muscle myosin heavy chain 9) [9]. As reviewed by Zhang et al., transferring CD163 alone is sufficient for conferring permissivity to a number of PRRSV non-permissive cells (including hamster, porcine and feline kidney cell lines) [3]. Furthermore, CD163 knockout pigs are resistant to PRRSV infection [10, 11]. These studies confirm that CD163 is an indispensable receptor.

Primary PAMs are the major target of PRRSV infection and are the best cell model for studying PRRSV biology. Acquiring primary PAMs is expensive, and PAMs cannot be reliably frozen for long-term storage and use. Several attempts to generate primary immortalized PAMs (iPAMs) using SV40 large T antigen [12] or human telomerase reverse transcriptase (hTERT) have been made [13]. iPAMs that were developed by SV40 large T antigen failed to mediate virus entry by disrupting CD163 expression [14]. Furthermore, the mechanism through which large T antigen regulated CD163 was unclear in these studies. Most importantly, though CD163 was recognized as an indispensable receptor, the correlation between the CD163 expression level and PRRSV infection remains unclear, which hinders our understanding of virus entry and pathogenesis.

Here, we successfully developed PRRSV-susceptible iPAMs by introducing SV40 large T antigen. We further found that the normal CD163 mRNA expression pattern was changed in a majority of the iPAMs, resulting in the failure of these cells to support viral replication. We isolated several iPAMs with varying CD163 abundances and demonstrated that only iPAMs with high CD163 abundance facilitate effective PRRSV infection.

## RESULTS

### Primary PAMs immortalized by introducing SV40 large T antigen fail to support PRRSV replication

**Figure 1:**
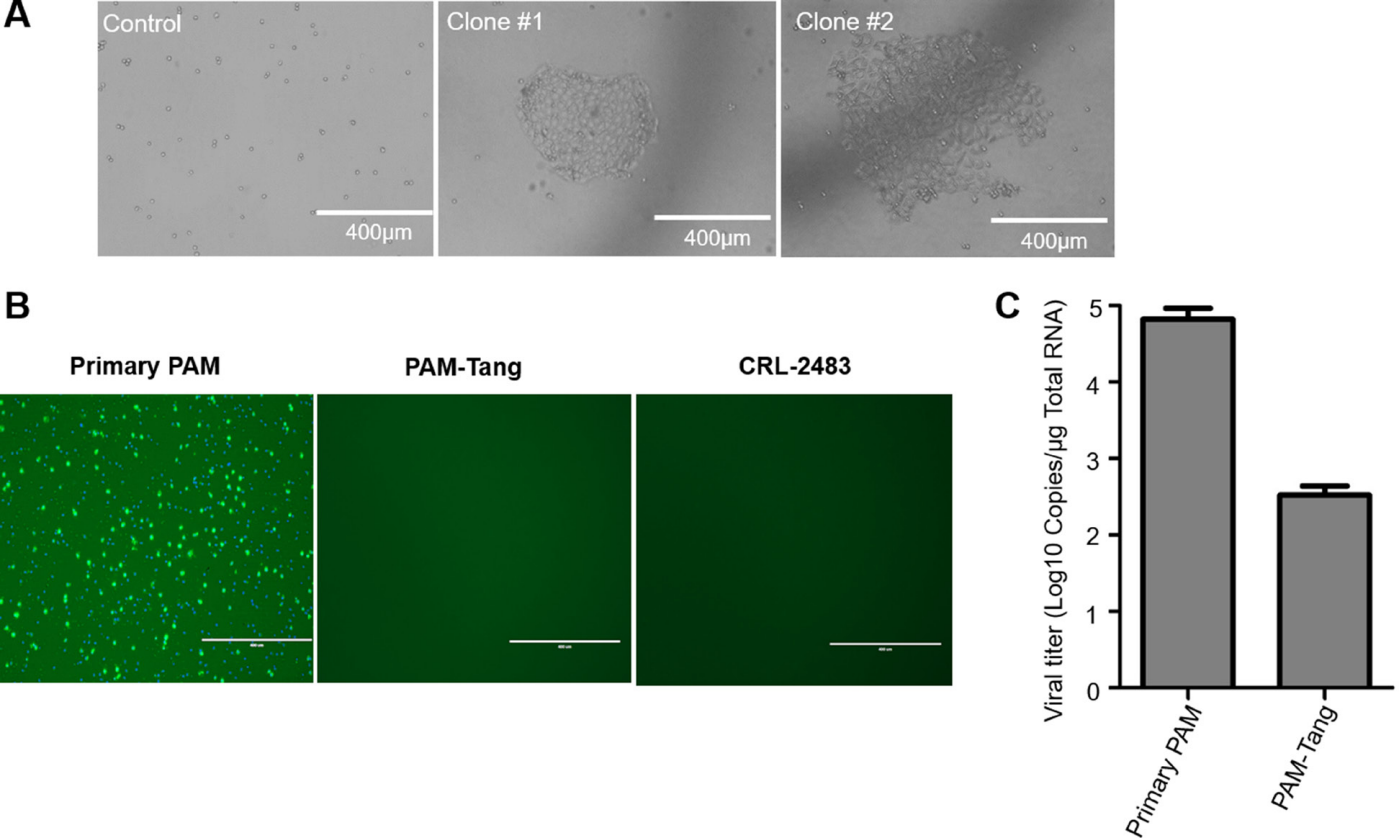
PAMs immortalized using SV40 large T antigen fail to support PRRSV replication (**A**) Primary PAMs were immortalized by introducing SV40 large T antigen, and two representative immortalized cell clones are shown. (**B**) Two immortalized iPAM cell lines, PAM-Tang and CRL-2483, failed to support PRRSV replication. Primary PAMs and immortalized cells were infected with PRRSV HuN4 at a multiplicity of infection (MOI) of 1. At 24 h post-infection, an immunofluorescence assay was performed, and the infected cells were then examined under an inverted fluorescence microscope. (**C**) PRRSV entry was blocked in iPAMs. The above experiments were performed three times, and a representative result is shown.

To generate iPAMs, we first isolated primary PAMs and introduced SV40 large T antigen using a murine leukemia virus (MLV) lentiviral vector. After primary PAM isolation, we determined the PAM purity via flow cytometry using CD14 and CD169 as primary PAM markers. The isolated cells were high-purity PAMs (Supplementary Figure 1). Then, the primary PAMs were plated at a low density in a 6-well plate, and SV40 large T antigen was introduced via an MLV-mediated lentiviral vector. As shown in Figure 1A, several typical cell islands were formed, which indicated that the PAMs were successfully immortalized. All of the immortalized clones were collected and designated PAM-Tang. To determine whether the PAM-Tang cells were permissive for PRRSV, we used PRRSV HuN4 to infect the PAM-Tang cells. At 12 h post-infection, an indirect immunofluorescence assay (IFA) was performed, and we found that PAM-Tang cells were non-permissive for PRRSV infection (Figure 1B). At the same time, primary PAMs serving as a positive control supported PRRSV replication. We also tested an iPAM line (CRL-2483) developed by another lab using large T antigen transformation [12], but neither iPAM cell line supported PRRSV replication (Figure 1B). CD163 has been reported to be an essential entry receptor for PRRSV; thus, we next tested whether the entry step was blocked in PAM-Tang cells. The results of this experiment indicated that PRRSV entry in PAM-Tang cells was indeed significantly blocked (Figure 1C).

### Few iPAM cells are CD163 positive

**Figure 2:**
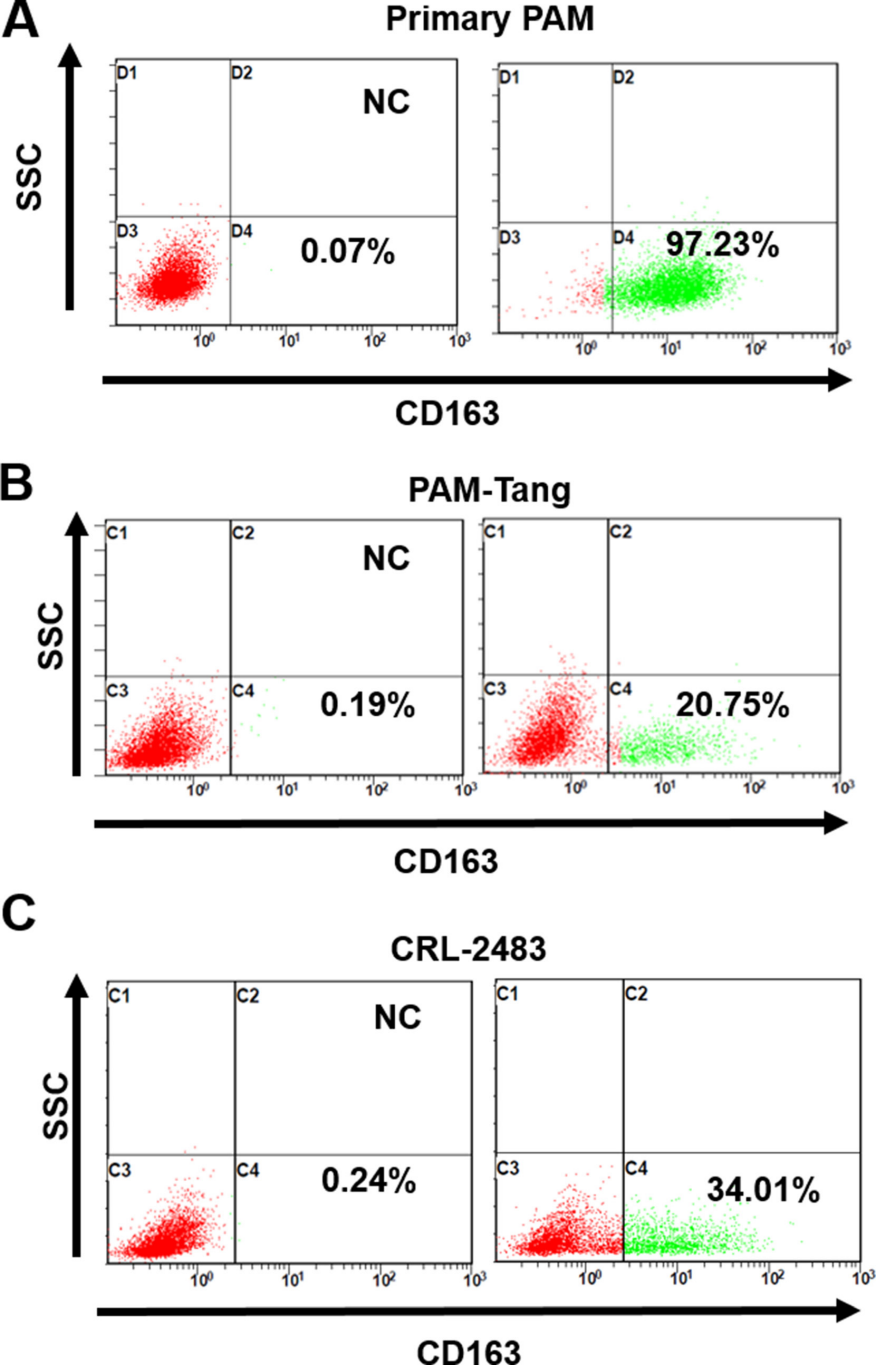
Few iPAM cells are CD163 positive (**A**) CD163 expression in primary PAM cells, (**B**) PAM-Tang cells and (**C**) CRL-2483 cells was identified at the protein level by flow cytometry. The above experiments were performed three times, and a representative result is shown.

A previous study indicated that CD163 was the sole determining factor for PRRSV entry in PAMs, which was supported by the evidence that introducing the CD163 gene alone into iPAMs was sufficient to restore PRRSV susceptibility [14]. Furthermore, as reviewed by Zhang et al., transferring CD163 is sufficient to confer permissivity to several types of non-PRRSV-permissive cells [3]. Here, we examined whether CD163 expression was abolished at the protein level in PAM-Tang cells using flow cytometric detection. As expected, in contrast to primary PAMs in which nearly all of the cells were CD163 positive (Figure 2A), only approximately 20.75% of PAM-Tang cells were CD163 positive, and 34.01% of CRL-2483 cells were CD163 positive (Figure 2B and 2C). These findings indicated that the CD163 expression pattern was altered in iPAMs.

### iPAMs mainly express a shorter form of CD163 via alternative splicing

**Figure 3:**
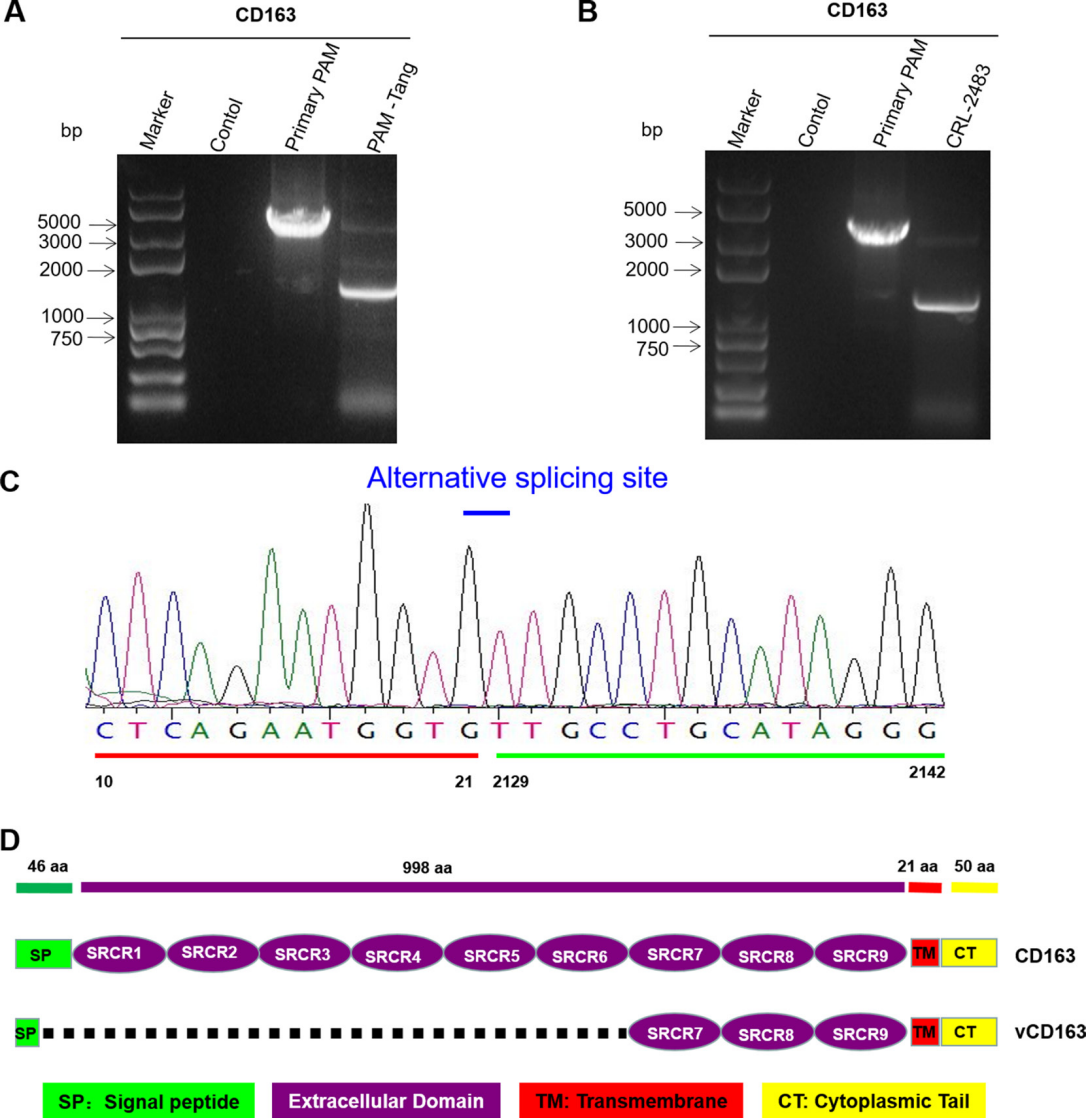
iPAMs mainly express short-form CD163 via alternative splicing (**A**) PAM-Tang cells mainly expressed short-form CD163. Total RNA was extracted from PAM-Tang cells, and reverse-transcription PCR was performed. This experiment was performed three times, and a representative result is shown. (**B**) CRL-2483 cells also mainly expressed short-form CD163. Total RNA was extracted from CRL-2483, and reverse-transcription PCR was performed. This experiment was performed three times, and a representative result is shown. (**C**) DNA sequencing of short-form CD163. (**D**) A schematic map of the short-form CD163 deletion compared with full-length CD163.

**Figure 4:**
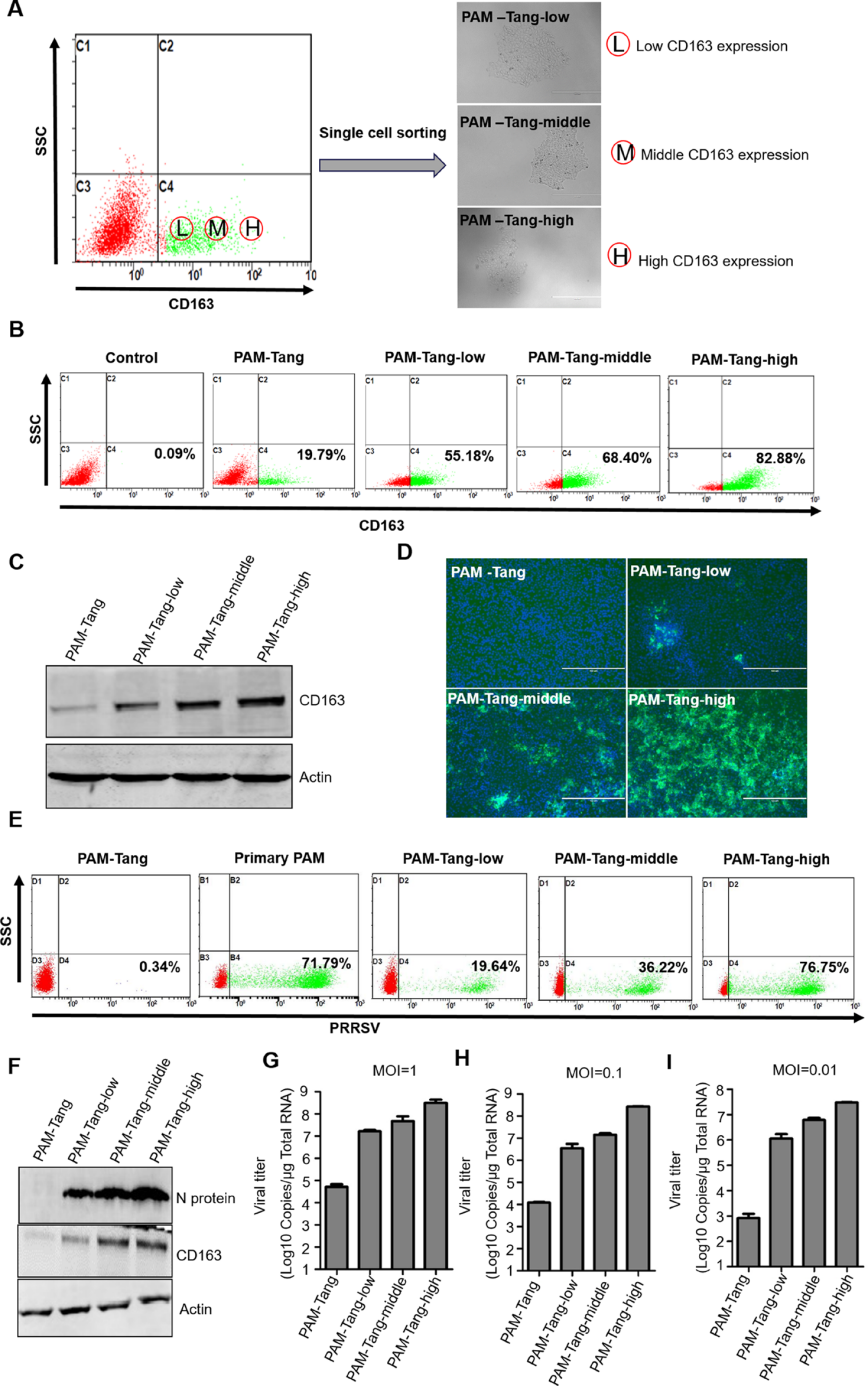
High levels of CD163 expression determine PRRSV replication (**A**) Three CD163 expression patterns (low, middle and high levels) were isolated using flow cytometry sorting technology. The representative isolated cell clones were designated PAM-Tang-low, PAM-Tang-middle and PAM-Tang-high, respectively. (**B**) The CD163 expression levels of PAM-Tang-low, PAM-Tang-middle and PAM-Tang-high cells were confirmed by flow cytometry and (**C**) Western blotting. (**D**) PAM-Tang, PAM-Tang-low, PAM-Tang-middle and PAM-Tang-high cells showed different susceptibilities to PRRSV infection. All immortalized cells were infected with PRRSV HuN4 at an MOI of 1. Five days post-infection, the infected cells were examined under an inverted fluorescence microscope and by (**E**) flow cytometry and (**F**) Western blotting. The above experiments were performed three times, and a representative result is shown for each. (**G**, **H** and **I**) Different cell lines were infected with different MOIs (1, 0.1 and 0.01), and 72 h post-infection, their viral titers were determined by real-time PCR.

Several aspects can influence cell surface receptors, such as alternative splicing [15], down regulation by microRNAs [16] or other cell pathways that act at the protein level [17]. We first detected CD163 expression at the RNA level, contrary to a previous report that indicated that CD163 expression was undetectable at the RNA level [14]. We found that CD163 mRNA was detectable in both primary PAMs and iPAMs (Figure 3A). However, the expression patterns differed between the primary PAMs and PAM-Tang cells. In primary PAMs, CD163 was expressed at its normal length, whereas a short form of approximately 1500 bp was observed in PAM-Tang cells (Figure 3A). Only very few CD163 RNA molecules were of normal length in PAM-Tang cells, explaining why only a few cells were CD163 positive. To test whether this difference was common for large T antigen-transformed iPAMs, we also examined CD163 expression in CRL-2483 cells, which similarly expressed two transcripts: a normal full-length CD163 and a short-form CD163 (Figure 3B). The majority of CD163 transcripts in the iPAMs was short-form CD163, and a limited level of full-length CD163 was detected (Figure 3B). We speculated that the difference in CD163 expression between iPAMs and primary PAMs was due to alternative splicing. To test this hypothesis, we cloned and sequenced the shorter form RNA transcript and found that a large fragment was absent in the short-form CD163 (Figure 3C). CD163 is a membrane-associated protein that consists of a signal peptide (SP), nine scavenger receptor cysteine-rich (SRCR) tandem repeats, a transmembrane motif (TM) and a cytoplasmic tail (CT). We further compared normal CD163 and short-form CD163 and demonstrated that the short CD163 mRNA lacks part of the SP and the first six SRCRs domains (SRCR1-SRCR6) (Figure 3D). This deletion results in frameshift mutations in which ORFs are misread and prematurely terminated (data not shown). Because the SRCR5 domain of CD163 is a critical domain for PRRSV entry [18, 19], the short-form CD163 transcript, which lacks SRCR5, does not support PRRSV entry. High levels of CD163 expression determine PRRSV replication. Although low levels of CD163 expression (from 20.75% to 34.01%) could be detected in both iPAM cell lines (Figure 2B and 2C), PRRSV still failed to replicate (Figure 1B), which raised the question of whether efficient PRRSV infection might require a high level of CD163 expression in target cells. To test this possibility, we isolated CD163-positive cells via flow cytometry sorting technology. Three CD163 expression patterns (low, middle and high levels of expression) were established to isolate single-cell derived clones (Figure 4A). The representative isolated cell clones were designated PAM-Tang-low, PAM-Tang-middle and PAM-Tang-high, respectively (Figure 4A). To confirm that the above cells were isolated based on their CD163 differences, we further detected CD163 levels by flow cytometry, and the results demonstrated that PAM-Tang-low, PAM-Tang-middle and PAM-Tang-high differed in their CD163 expression levels, consistent with our expectations (Figure 4B). We also detected CD163 expression at the protein level, further confirming that the CD163 levels in these cell lines were as expected (Figure 4C). Next, we explored the relationship between PRRSV replication and CD163 expression level. After infection with PRRSV-HuN4, we found that the PAM-Tang-low line, which contained 55.18% CD163-positive cells, showed limited PRRSV replication; however, PRRSV replicated efficiently in both the PAM-Tang-middle (68.4% CD163-positive cells) and PAM-Tang-high lines (82.88% CD163-positive cells) (Figure 4D). We further analyzed the infection ratios of these different cells by flow cytometry and found that the number of infected cells increased with increasing CD163 abundance (Figure 4E); the same conclusion was reached at the viral protein level (Figure 4F). Finally, we evaluated the susceptibilities of these cell lines by viral titer, infecting them with different multiplicities of infection (MOIs; 1, 0.1 and 0.01); 72 h post-infection, viral RNA was extracted and quantified by real-time PCR. The results indicated that PRRSV replicated most efficiently in PAM-Tang-high cells (Figure 4G, 4H and 4L). To test whether this phenomenon was common to other PRRSV strains, we also tested an NADC30-like strain (SC-D) and found that it replicated more efficiently in CD163-high cells than in CD163-low cells (Supplementary Figure 2).

**Figure 5:**
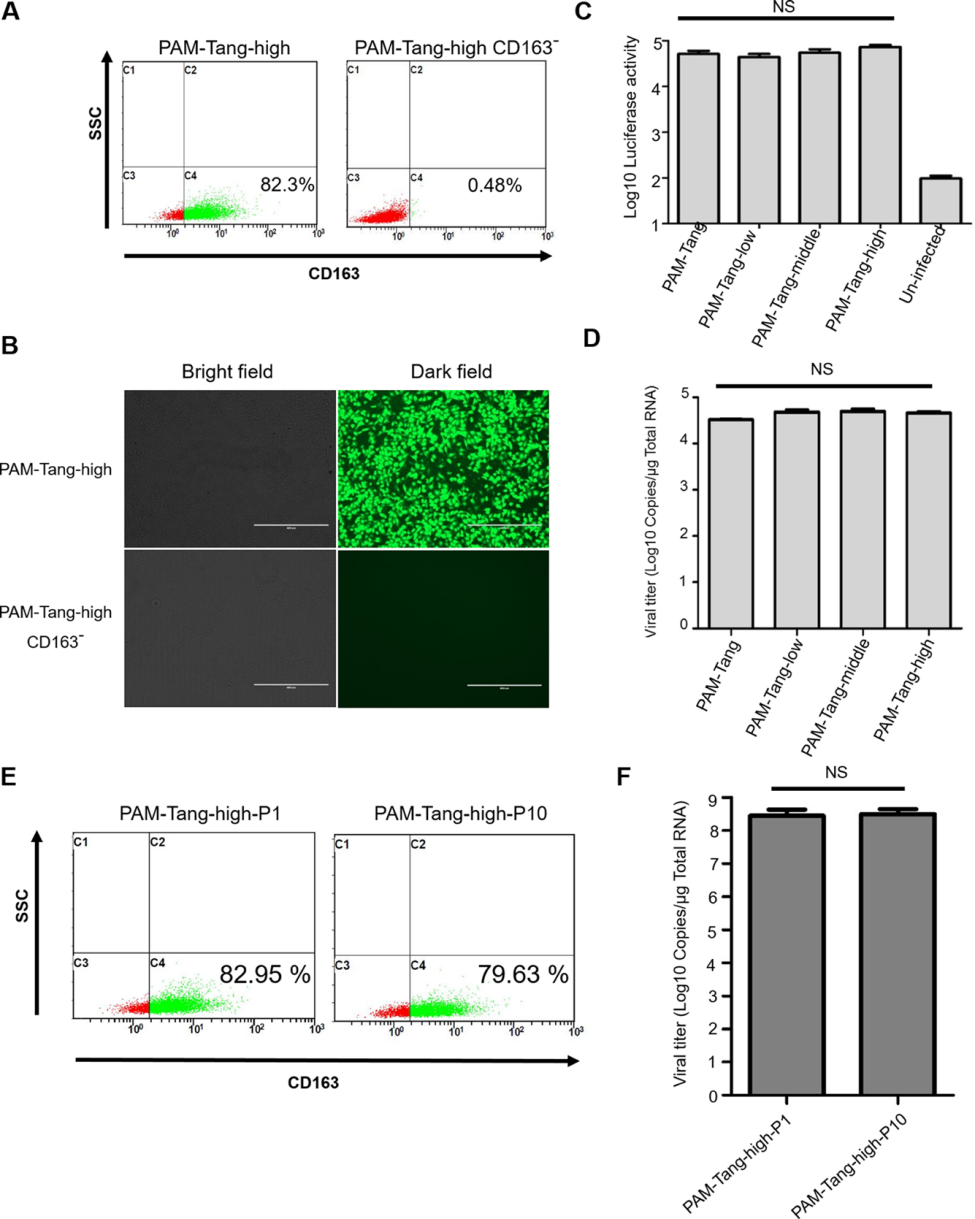
CD163 is critical for PRRSV replication (**A**) CD 163-negative cells isolated from PAM-Tang-high cells by flow cytometry. (**B**) CD163 depletion in PAM-Tang-high sufficiently blocks PRRSV replication. (**C**) PRV infection was not influenced by CD163 abundance. A firefly luciferase-tagged PRV (MOI = 1) was utilized to infect different cell lines, and luciferase activity was evaluated at 24 h post-infection. (**D**) PRRSV attachment was not influenced by CD163 abundance. (**E**) The CD163 level of PAM-Tang-high cells did not significantly change during the course of 10 passages. (**F**) Cellular susceptibility to PRRSV did not significantly change during the course of 10 passages. PAM-Tang-high P1 and PAM-Tang-high P10 were infected with 1 MOI of PRRSV HuN4, and their viral titers were determined by RT-PCR at 72 h post-infection.

To further support this conclusion, we depleted CD163 expression in PAM-Tang-high cells to exclude the possibility that other factors were contributing to their susceptibility to PRRSV infection. We used CD 163-negative cells isolated from PAM-Tang-high cells by flow cytometry (Figure 5A) to perform an infection assay, and the results demonstrated that CD163 depletion in PAM-Tang-high is sufficient to block PRRSV replication (Figure 5B). To test whether this effect was PRRSV-specific, we also infected different cell lines with a swine DNA virus and a firefly luciferase-tagged Pseudorabies virus (PRV) [20]. At 24 h post-infection, luciferase activity was evaluated, and the data suggested that PRV infection was not influenced by CD163 abundance (Figure 5C). As increasing the CD163 abundance enhances the susceptibility of iPAMs to PRRSV, we next tested whether CD163 influences PRRSV entry during cell attachment. We performed a virus attachment assay and determined that virus attachment was not influenced by CD163 abundance (Figure 5D). We then tested the genetic stability of CD163 in PAM-Tang-high cells during the course of passage. PAM-Tang-high cells were passaged 10 times, and the CD163 levels of the 10 generations were evaluated. We found that over the course of 10 passages, the CD163 levels in PAM-Tang-high cells did not significantly change (Figure 5E). Furthermore, cellular susceptibility to PRRSV was evaluated and also remained unchanged over the course of 10 passages (Figure 5F). The above findings support the idea that effective PRRSV infection requires high levels of constitutive CD163 expression.

### iPAM expression patterns change significantly

**Figure 6:**
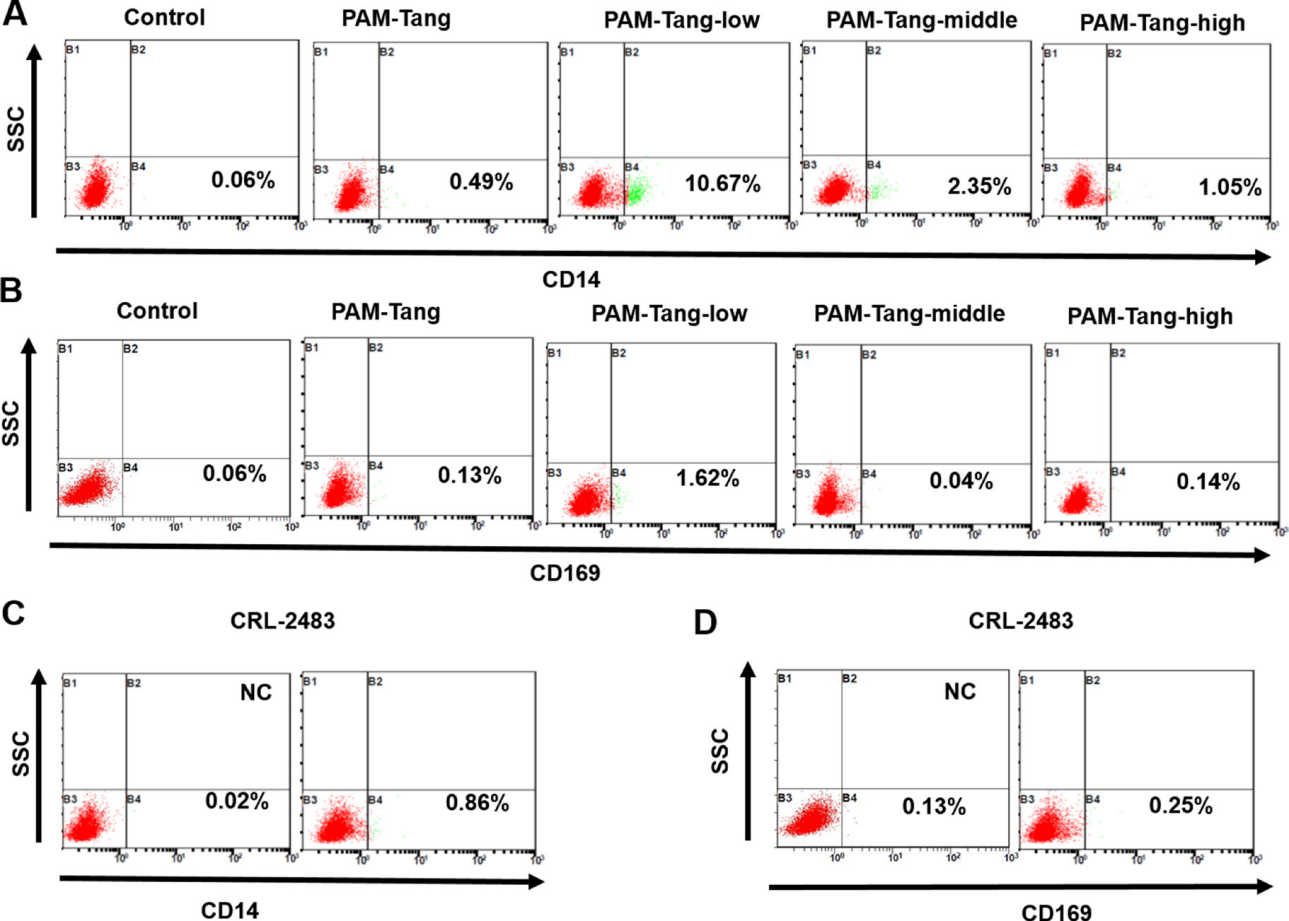
iPAM expression patterns change significantly (**A**) CD14 expression profiles of different cell lines were evaluated by flow cytometry. (**B**) CD169 expression profiles of different cell lines were evaluated by flow cytometry. (**C**) CD14 expression in CRL-2483 cells was evaluated by flow cytometry. (**D**) CD169 expression in CRL-2483 cells was evaluated by flow cytometry. The above experiments were performed three times, and a representative result is shown for each.

The above data indicated that the CD163 expression pattern was altered in the iPAMs. Next, we determined whether other markers of primary PAMs were also influenced. We first detected CD14, a macrophage marker. A very limited number of PAM-Tang cells expressed CD14, and only 10.67% of PAM-Tang-low cells were CD14 positive (Figure 6A). We further detected an attachment receptor for PRRSV, CD169, and its expression was also disrupted in all of the iPAMs (Figure 6B), which further supports the hypothesis that CD163 is the determinant receptor for PRRSV. To determine whether these expression patterns were unique to PAM-Tang cells, we likewise examined the CRL-2483 cell line and found that the CD14 and CD169 expression levels were almost completely lost (Figure 6C and 6D). To summarize, iPAMs change significantly, from cell morphology (Figure 1A) to molecular markers.

## DISCUSSION

The MA-104 (African green monkey kidney) and MARC-145 (a derivative of MA-104) cell lines are susceptible to PRRSV infection and have both been used widely for PRRSV studies [21]. However, both of these cell lines are derived from the kidney of the African green monkey, and whether monkey-derived cells reflect the biological properties of porcine target cells is often questioned. Primary PAMs are the major target of PRRSV *in vivo*, and they are a suitable cell model for elucidating PRRSV biology. Primary PAMs can be transformed by SV40 large T antigen or hTERT [12, 13]. SV40 large T antigen targets multiple cellular pathways to elicit cellular transformation, and increasing evidence has demonstrated that large T antigen exerts its effects by inhibiting tumor suppressors in both the p53 and Rb families [22]. However, it is not yet clear whether the Rb and p53 proteins are the only targets of large T antigen-transformed cells; the existence of additional targets should be further explored [22].

In a previous report, iPAMs developed using SV40 large T antigen influenced CD163 expression at both the mRNA and protein levels, which subsequently prevented virus entry [12]. In our study, we found that non-susceptible iPAMs expressed CD163 at the RNA level, mainly as short-form transcripts. The previous study did not detect CD163 RNA, possibly due to the primers they used. We speculate that they isolated their iPAMs using a traditional limited dilution assay, which failed to efficiently isolate limited CD163-positive iPAMs and may be the reason for their failure to immortalize PAMs that were susceptible to PRRSV. In the present study, we used flow cytometry sorting technology to isolate CD163-positive iPAMs. The majority of iPAMs failed to express full-length CD163, possibly due to changes in their DNA transcription patterns caused by the large T antigen. Furthermore, the large T antigen integration site(s) may also influence DNA transcription. These potential explanations need to be explored further. Whether the CD163 expression pattern is directly or indirectly influenced by large T antigen also needs to be investigated.

Fortunately, we were able to isolate several iPAMs with different CD163 expression levels in the present study, which allowed us to investigate the correlations between PRRSV infection and CD163 abundance. We demonstrated that the CD163 expression level in a target cell must reach or exceed an infection initiated threshold for PRRSV to establish a successful infection. This situation may be critical for PRRSV inter-species transmission. A previous study showed that CD163 from dogs, mice, monkeys and humans can serve as PRRSV entry receptors when transfected into non-susceptible hamster, porcine and feline kidney cell lines [23]. Why are the original CD163-expressing donor cells (with the exception of MARC-145) not themselves PRRSV-permissive? Our above data indicate that a high CD163 expression level is necessary for efficient PRRSV replication, which may explain this issue. The fact that the original CD163-expressing donor cells are not themselves PRRSV-permissive may due to the low expression levels of CD163 in the corresponding cell lines. For example, monkey-derived Vero cells were able to bind and internalize PRRSV, but the virus could not establish effective infection, a situation that can be reversed by the fusion of PRRSV to Vero cells using polyethylene glycol (PEG) [24]. Furthermore, human-derived HEK293 cells that stably express a high level of swine CD163 were susceptible to PRRSV infection [25]. These studies indicate that once PRRSV can enter the target cells of other species, including human cells, it will replicate efficiently. However, the exact initiation threshold of targeted cells must be acquired by analyzing several iPAMs with CD163 expression levels from 19.79% to 55.18%, and our results in Figure 2C and Figure 1B show that CRL-2483 cells with a CD163 expression level of 34% were unable to support PRRSV replication. This finding indicated that the PRRSV infection threshold was between 34% and 55.18%. Unfortunately, we failed to isolated iPAMs with CD163 expression levels between 34% and 55.18%.

**Figure 7:**
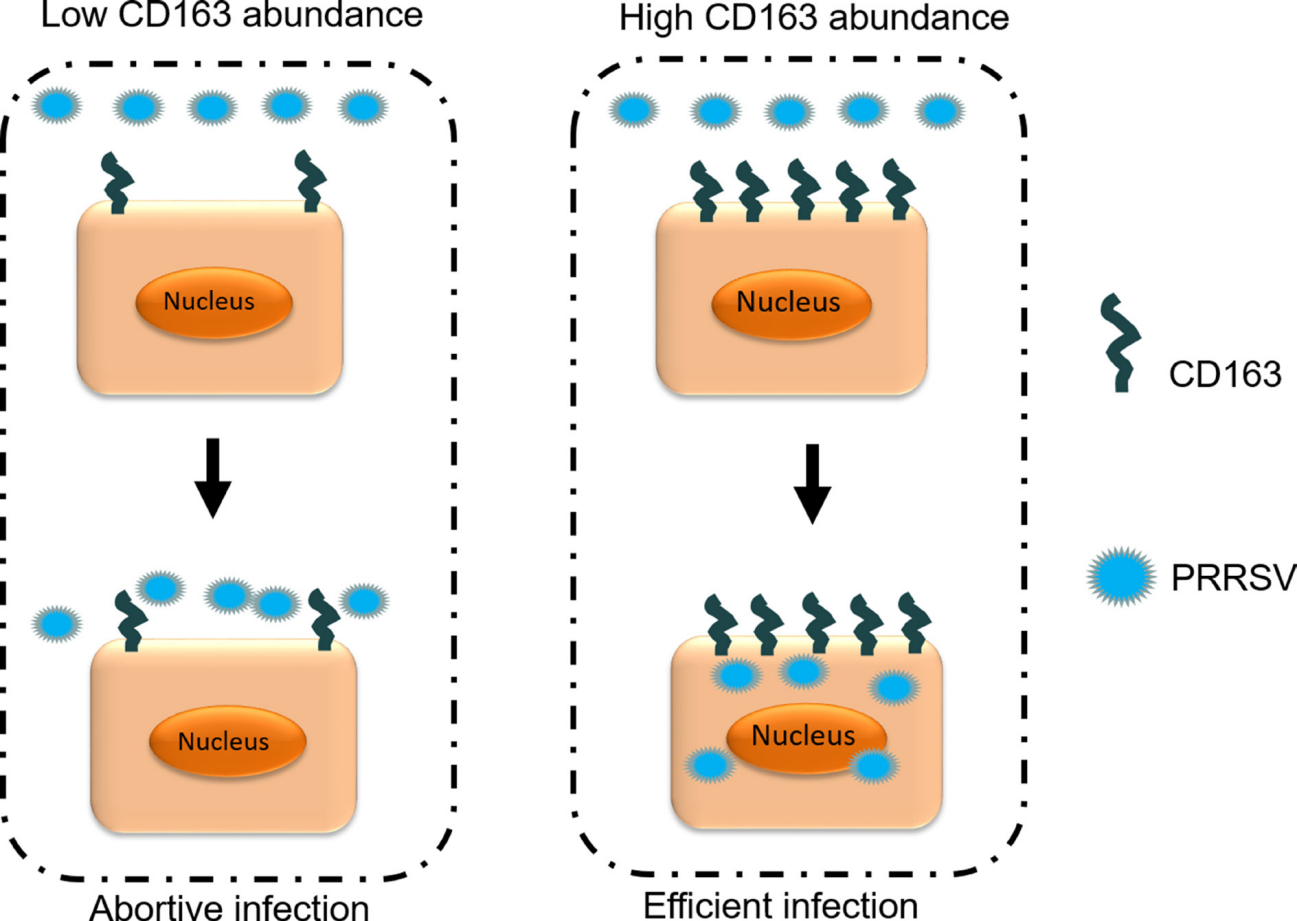
Relationship between CD163 abundance and viral infection Increasing the abundance of CD163 enhanced cellular susceptibility to PRRSV. Efficient PRRSV infection required high levels of constitutive CD163 expression.

In conclusion, we successfully developed PRRSV-susceptible iPAM cell lines by introducing the large T antigen, demonstrating that it hampers normal CD163 expression in a majority of iPAMs. We also isolated several iPAMs that expressed different levels of CD163 and established that the CD163 expression levels were critical for PRRSV infectivity: increasing the abundance of CD163 enhances cellular susceptibility to PRRSV. Efficient PRRSV infection requires high levels of constitutive CD163 expression, which is illustrated in Figure 7.

## MATERIALS AND METHODS

### Cells and viruses

Primary PAMs were isolated from 4-wk-old specific pathogen-free (SPF) piglets as previously described [26, 27]. Animal experiments were approved by the Animal Ethics Committee at the institute and were performed in accordance with animal use ethical guidelines and approved protocols. Primary PAMs, iPAM cell lines and plat E cells were all maintained in Dulbecco’s modified Eagle medium (DMEM) (Invitrogen) supplemented with 10% fetal bovine serum (FBS; Gibco) and 1% antibiotics (penicillin and streptomycin) at 37°C with a humidified atmosphere of 5% CO_2_. The highly pathogenic PRRSV strain HuN4 that we used was described previously [28]. PRRSV-SC-D is a NADC30-like strain that was isolated in our lab (unpublished data). The firefly luciferase-tagged Pseudorabies virus (PRV) was described previously [20].

### Lentivirus packaging

Large T antigen was cloned from HEK293T cells and then inserted into the lentiviral transfer plasmid pSFG using the NcoI cloning site. A total of 7 × 10^5^ plat E cells were cultured on a 60-mm culture dish, and when each dish was at 60% confluence, it was transfected with 18 μg of pSFG transfer vector, 12 μg of Gag-pol packaging vector and 6 μg of VSV-G using a calcium phosphate transfection reagent. Viruses were collected 48 h after transfection and were centrifuged at 10,000 × g for 2 min to remove cell debris. The packaged lentivirus was stored at –80°C.

### Primary PAM immortalization

Primary PAMs were infected with 4.5 ml of lentivirus in a 15-ml centrifuge tube, followed by centrifugation at 1500 × g for 2 h at room temperature. The supernatant was discarded, and the cell pellet was resuspended thoroughly in 5 ml of fresh medium containing 10% FBS and 1% antibiotics (penicillin and streptomycin) and then cultivated on 6-well plates until the primary PAMs were immortalized.

### Flow cytometry analysis and cell sorting

Different cells and cell lines were seeded in 6-well plates at a density of 6×10^5^ cells per well; after 12∼18 h, the cells were washed three times with PBS and subsequently detached with trypsin-EDTA. A total of 6 × 10^5^ cells were resuspended with PBS, collected into 2-ml EP tubes, and incubated with 10 µl of PE-conjugated mouse anti-porcine CD163 mAb (1:200), a PE-labeled antibody (Abcam), for 30 min at room temperature according to the manufacturer’s instructions. Then, the cells were gently washed twice with PBS to remove the unbound antibody. Meanwhile, the negative group was stained with a mouse IgG1 as the isotype control. CD14 and CD169 were analyzed using FITC- and APC-conjugated antibodies (Abcam), respectively. For PRRSV infection analysis, different cell lines were infected with PRRSV HuN4 (Multiplicity of infection (MOI) = 1), and 72 h post-infection, the cells were washed three times with PBS and subsequently detached with trypsin-EDTA. The cells were then fixed/permeabilized and stained with PRRSV N specific antibody, and labeled FITC was used as the secondary antibody. Flow cytometric analyses and sorting were performed on an FACS Aria instrument (BD Biosciences).

### Western blot analysis and indirect immunofluorescence assay (IFA)

Western blots were performed as previously described [29]. IFA assay was similar to a previously report by using indicated antibodies [30].

### Alternative splicing analysis

Total RNA was extracted from primary PAMs and iPAMs using an RNeasy Plus Mini kit (Takara). Reverse Transcriptase M-MLV (RNase H-) (TaKaRa) was used to make cDNA, which was then amplified by KOD DNA polymerase (Takara) using primers (F: 5′-GTAATAATACAAGAAGATT-3′ and R: 5′-TCATTGTACTTCAGAGTGGTC-3′). Gel electrophoresis was used to analyze the obtained PCR products, which were then purified with a Gel Extraction Kit (OMEGA) according to the manufacturer’s instructions. The purified PCR products were further analyzed by DNA sequencing.

### Virus attachment and entry assay

Different cell lines were plated in 12-well plates (5 × 10^5^ cells). When the cells grew into a monolayer, they were kept at 4°C for 30 min and then inoculated with PRRSV HuN4 (MOI = 1) at 4°C for 2 h. The cells were washed three times with cold PBS to remove unbound virus. Total RNA was extracted from the cells using the methods described above, and 1 μg of total RNA was used for cDNA synthesis. A real-time reverse-transcription-PCR (RT-PCR) assay was used to quantify attached virus particles as described previously [31]. For the PRRSV entry assay, different cell lines were plated in 12-well plates (5 × 10^5^ cells). When the cells grew into a monolayer, the cells were kept at 4°C for 30 min and then inoculated with PRRSV HuN4 (MOI = 1) at 4°C for 2 h. The cells were washed three times with cold PBS to remove unbound virus and further incubated at 37°C for 2 h. Total RNA was extracted and RT-PCR was performed as described above.

### Statistical analysis

Values are expressed as the means ± the SD. The data were analyzed with Student *t* tests. A *P* value of < 0.05 was considered significant.

## SUPPLEMENTARY MATERIALS FIGURES



## Abbreviations

PRRSV: porcine reproductive and respiratory syndrome virus
PAM: porcine alveolar macrophages
