# Supplementary material for: Porcine alveolar macrophage CD163 abundance is a pivotal switch for porcine reproductive and respiratory syndrome virus infection
Source: Oncotarget. 2018 Jan 6;9(15):12174–85. doi: 10.18632/oncotarget.24040 (PMC5844737; doi:10.18632/oncotarget.24040)
Supplement: Supplementary file 1 [file oncotarget-09-12174-s001.pdf]

Porcine alveolar macrophage CD163 abundance is a pivotal switch for porcine reproductive and respiratory syndrome virus infection

SUPPLEMENTARY MATERIALS

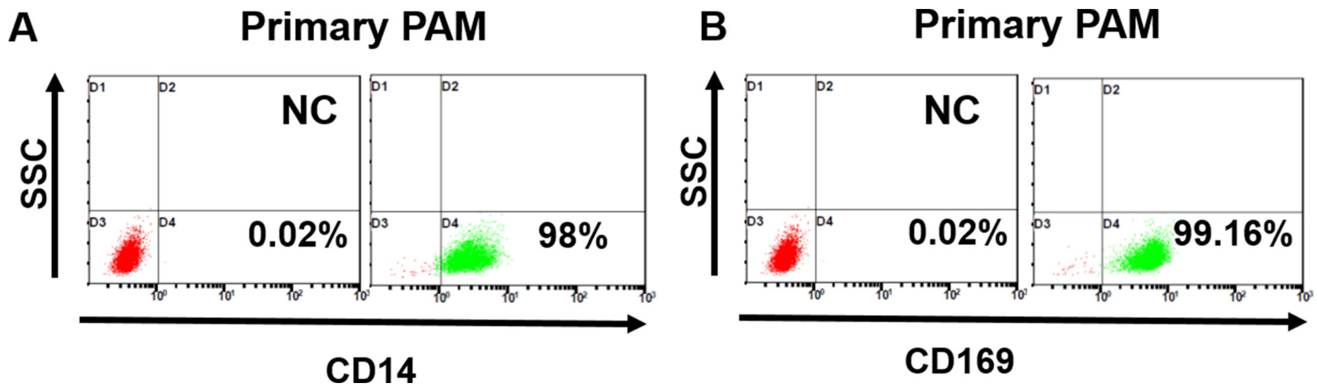

Supplementary Figure 1: Identification of primary PAM purification with indicated markers.

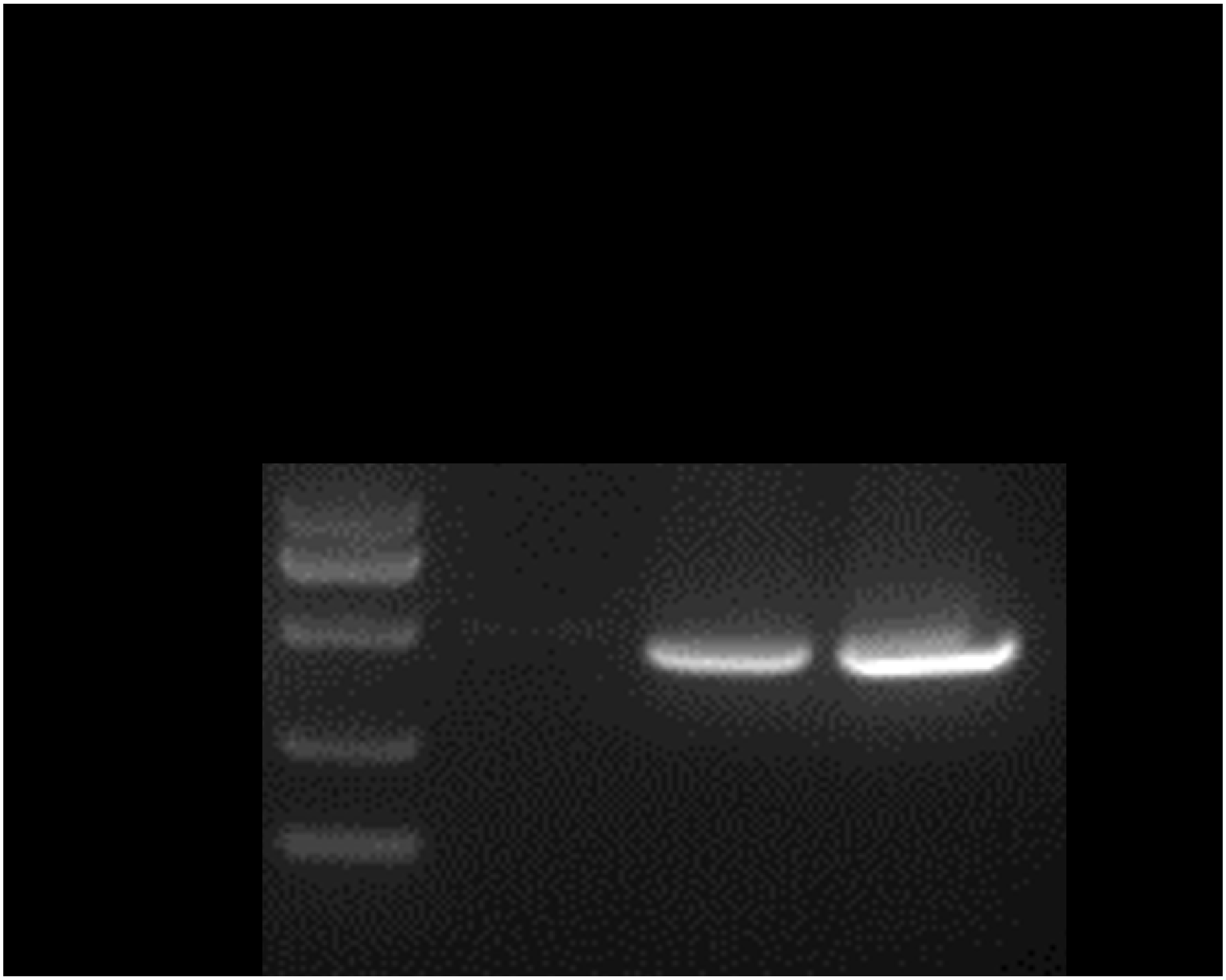

Supplementary Figure 2: Infection of iPAMs by NADC30-like PRRSV strain.
